# 

**DOI:** 10.1038/sj.bjc.6600376

**Published:** 2002-01-02


CONCLUSION: The majority of these patients had bulky disease which could not have received immediate RT with curative intent, on account of risk of spinal cord damage, even with concomitant chemotherapy. Induction chemotherapy allows down-staging of disease and we feel should not be discontinued, though our protocol now also includes concomitant cis-or carboplatin with RT.

**P99**

**THE EFFECT OF PHYTOESTROGENS ON PROSTATE CANCER CELLS *IN VITRO*.**

Colm Morrissey*, R. William G. Watson and John M. Fitzpatrick
Department of Surgery, Conway Institute of Biomolecular and Biomedical Research, University College Dublin, Mater Misericordiae Hospital, Dublin, Ireland.

Androgen independence represents the most non-treatable form of prostate cancer. In the past, advanced stage prostate cancer was treated using the synthetic oestrogen diethylstilbestrol, reducing serum testosterone levels by negative feedback on the pituitary-gonadal axis. Phytoestrogens are non-steroidal plant-derived compounds possessing oestrongenic activity. We hypothesized that these compounds would increase the effects of the anti-androgens in their ability to induce cell death. PC-3 (malignant androgen independent) LNCaP (malignant androgen sensitive) and RWPE-1 (benign androgen sensitive) cells were treated with or without an extract of *Belacanda Chinensis,* two purified phytoestrogens derived from this extract; irigenin and tectorigenin and the anti-androgen biclutamide, and assessed for their effects on cell number and apoptosis using the crystal violet assay and propidium iodide DNA staining assays, respectively. The phytoestrogens (50-100 μM) and biclutamide (10-50 μM) alone decrease cell number in all three cell lines. When the phytoestrogens (50 μM) are used in combination with biclutamide (10 μM) there is no statistical difference in the loss of cell number in the RWPE-1 and LNCaP cell lines. However, in the androgen insensitive PC-3 cell line all three phytoestrogens showed a statistically significant decrease in cell number when treated with all three compounds in conjunction with biclutamide with P values of 0.017, 0.023 and 0.049 for the extract, irigenin and tectorigenin respectively. The phytoestrogen effects are increased when used in combination with biclutamide in the PC-3 cell line. Biclutamide induced apoptosis in a dose dependent manner, but the phytoestrogens had no effect. These *in vitro* studies will lead to a better understanding of the mechanism of action of the phytoestrogens and their possible use in prostate cancer prevention or management.

**P100**

**A PHASE I/II STUDY OF GEMCITABINE (G) AND CISPLATIN (C) IN METASTATIC OR RELAPSED TRANSITIONAL CELL CARCINOMA IN AN OUTPATIENT SETTING.**

Syed A. Hussain*[1, 2], Ian Geh[2], Peter Riley[2], Nicholas D. James[1, 2] Cancer Research UK Institute For Cancer Studies[1] Queen Elizabeth Hospital[2] Birmingham, United Kingdom

**Introduction:** A randomised phase III trial of MVAC vs. GC (G 1000mg/m2 d1, 8+15, C 70mg/m2 d2, 28 d cycle) (JCO 2000 17:3068-3077) showed that, with the same efficacy, GC had a better toxicity profile. The most important haematological toxicities of GC were noticed on d15 resulting in dose adjustments in 37% of cycles. To optimise drug delivery and minimise platinum related toxicity, we are exploring administration of GC on a 21 day cycle with G and C split between D1 and D8 in a phase I/II mixed dose escalation. Primary objectives are definition of maximum tolerated dose (MTD) and toxicity; secondary endpoints are response rate, progression free and overall survival.

**Methods:** Patients with locally advanced, relapsed or metastatic disease are eligible. ECOG PS 0-2, adequate bone marrow, liver function, GFR > 40mls/min. Planned dose escalation G/C 1000/35, 1100/35, 1200/35, 1200/45 mg/m2 treated days 1 & 8 of a 21 day cycle.

**Results:** 15 patients have been enrolled (9 M, 6 F, age 41-79, median 64 y); 12 N+ disease, 8 M1 (1 bone, 1 lung plus bone, 1 lung plus liver, 1 liver, 3 lung, 1 psoas and pre-sacral tumour deposit); 2 had previous cystectomy; 1 had a open and close laparotomy, 5 had previous radical RT (2 synchronous chemoradiation); 2 previous CMV chemotherapy; 8/15 had GFR of 40-60mls/min. Dose limiting toxicity (DLT) was haematological (Grade IV thrombocytopenia) and was reached at dose level 2. The extended phase II study is continuing at dose level 1 (1 level below the DLT) as per protocol. At dose level 1 there was 1 grade IV episode (GI perforation), toxicity was otherwise grade III (anaemia 1/4, neutropenia 1/4, thrombocytopenia 1/4). At dose level 2 more severe haematological toxicity was encountered (anaemia 1/9, neutropenia 4/9 (1 grade IV), thrombocytopenia 5/9 (3 grade IV)) thereby defining the MTD. Non-haematological toxicity included 1 GI perforation and 1 GI bleed (both G IV). Of 55 cycles administered, we observed platelets <100 d15 in 22/55 cycles, neutrophils <0.5 or plat<50 in 9/55 cycles; but only 2/55 cycles (in the same patient) were deferred at day 21 for haematological toxicity. Renal functions have remained stable on this regimen. Median **dose intensity** delivered was 96% (Range 78% -100%). There were 2 early deaths (GI perforation on steroids as anti emetic; GI bleed on warfarin for DVT secondary to massive pelvic lymphadenopathy, found to have pyloric ulcer) and were not assessable for response but included in the efficacy analysis. Two patients are enrolled in the phase II study, 9 pts have undergone response assessment of which 3 had CR, 4 PR to give an interim overall response rate of 7/11 (64%) for the phase I part of the trial.

**Conclusion:** The MTD of this split dose schedule has been defined and the regimen is deliverable on an outpatient basis without the need for inpatient hydration. The schedule has substantial activity even in pre-treated patients with renal function below that conventionally included in protocols with this combination and the phase II trial is ongoing.

**Key words:** Bladder Cancer Gemcitabine and Cisplatin Out-patient treatment

**P101**

**THE EFFECT OF COMBINATION GEMCITABINE AND PHOTODYNAMIC THERAPY ON CLONOGENIC SURVIVAL IN BLADDER CANCER CELL LINES IN VITRO**

C Briggs[1,2], JV Moore[2], NW Clarke[1]
[1]Department of Urology, Hope Hospital, Salford
[2]Paterson Institute for Cancer Research, Christie Hospital, Manchester

**Introduction**

Gemcitabine is a cytosine analogue, which has radiosensitising properties and antitumour activity in a variety of solid tumours. Its effects in combination with photodynamic therapy have not previously been reported. The aim of this study was to determine the effect of combination treatment with gemcitabine and aminolaevulinic acid-mediated photodynamic therapy (ALA PDT) in two related bladder TCC cell lines.

**Materials and Methods**

Two related bladder cancer cell lines were studied: the radioresistant parent line MGH-U1 and its radio-resistant clone, S40b. Standard clonogenic assays were performed following treatment with ALA PDT alone (5mM ALA for 24 hours prior to PDT) or in combination with gemcitabine (0.01μM +5mM ALA for 24 hours before PDT).

**Results**

Clonogenic survival assays showed that both cell lines were sensitive to PDT alone, though MGH-U1 was significantly more-so than S40b (p<0.001). This was explained by a 4-fold greater level of protoporphyrin IX (PpIX) fluorescence in the MGH-U1 cell line. Prior exposure to gemcitabine produced a 2-fold decrease in cell survival in the radio-resistant cell line, but caused the radiosensitive S40b cell line to become relatively resistant to PDT under the same conditions (p<0.001).

**Conclusions**

Gemcitabine increases the sensitivity of TCC to ALA PDT, but a differential effect is seen in radiosensitive and radio-resistant cells. Work continues to establish mechanisms accounting for this response, but a role for combination treatment with radiosensitisers and PDT in superficial bladder cancer treatment is suggested.

© 2002 Cancer Research UK

**P102**

**MANAGEMENT OF PATIENTS WITH ADVANCED GERM CELL TUMOURS (GCT): IS THERE A ROLE FOR POMB-ACE?**

N. Bhala, J. George, J.M. Coleman, C.R. Radstone, J.M. Horsman, B.W. Hancock, M.Q. Hatton, R.E. Coleman*.
YCR Academic Unit of Clinical Oncology, Weston Park Hospital, Sheffield, United Kingdom.

**Aims** Germ cell tumours (GCT) are the commonest malignancies in young men and overall survival has been increased with the introduction of cisplatin-based therapy. Previous trials have indicated BEP (bleomycin, etoposide and cisplatin) as the optimum treatment, although some centres advocate the use of the alternating regimen POMB/ACE (cisplatin, vincristine, methotrexate, bleomycin & actinomycin D, cyclophosphamide and etoposide) in more advanced metastatic disease. We undertook a study of patients treated at Weston Park Hospital, Sheffield (WPH) and retrospectively analysed the survival and management of this population.
**Methods** Patient information was retrieved using the Trent Testicular Tumour Registry and from patient notes. This included all patients with Royal Marsden Hospital Stage II, III and IV disease and those with Stage I disease that relapsed. These data were then used to produce survival curves and the different chemotherapy regimens used were compared with regard to efficacy and toxicity.
**Results** 178 NSGCT and 71 seminoma patients were identified. Survival curves yielded outcomes that were similar to the IGCCCG[1] classification for the NSGCT good (Five year survival 95% vs. 92%), and intermediate (5 year survival 82% vs. 80%) prognoses groups. The survival of the poor prognosis group was markedly better at WPH (5 year survival 57% vs. 48%). There was a higher frequency of severe side effects with POMB/ACE.
Conclusions Survival and disease progression rates at this single institution were at least as good as those reported in the series published by IGCCCG[1] and better in the poor prognosis group. In the latter, the principal difference is the use of the POMB/ACE chemotherapy regimen as opposed to the standard BEP regimen. We would recommend a randomised comparison of BEP and POMB/ACE to validate this.

[1] International Germ Cell Cancer Collaborative Group. International Germ Cell Consensus Classification: A Prognostic Factor-Based Staging System for Metastatic Germ Cell Cancers. J Clin Oncol 1997; 15(2): 594-603.

**P103**

**TRENDS IN SURVIVAL OF PATIENTS DIAGNOSED WITH PRIMARY TESTICULAR TUMOURS IN LEICESTERSHIRE: A 15-YEAR EXPERIENCE (1983 – 1997)**

S.Santhanam*, M. Decatris, F.J.Madden. Dept. of Oncology, University Hospitals of Leicester. Leicester.

**AIM:** A retrospective analysis of all primary Testicular tumour patients treated at the University hospitals of Leicester between 1983 and 1997.
**METHODS:** 328 consecutive primary Testicular tumour patients (excluding lymphomas) diagnosed between 1983 and 1997 were included. Use of various treatment modalities, which influences the long-term treatment related sequelae and overall survival (OS) rates were analysed. To analyse the trends in survival, the 5-year OS of patients diagnosed between 1983 - 1990 (Cohort A) is compared with those diagnosed between 1991- 1997 (Cohort B). Statistical computations were performed using StatView® for Windows, (SAS Institute Inc., USA).
**RESULTS:** Overall, 104 patients had primary chemotherapy (CT), 158 had primary radiotherapy (RT), [of these 9 had combined therapy (RT + CT)], 69 were managed by active surveillance. Treatment data was missing for 6 patients. With a mean follow-up period of 72 months (median 62 months), the 5-yr and 10-yr OS for all patients was 92% and 88% respectively. The 5-yr OS for Cohort A and Cohort B was 90% and 94% respectively ($p > 0.1$). There were no significant differences in distribution of age, stage and histology between the two cohorts ($p > 0.26$, $> 0.70$, $> 0.66$ respectively). The number of patients treated by primary CT, primary RT and active surveillance remained stable over this study period. (Cohort A: 33%, 41%, 20%; Cohort B: 31%, 48%, 23%, respectively; $p > 0.62$). The number of patients treated by BEP (Bleomycin, Etoposide, Cisplatin) regime did not vary during this period. (Cohort A: 82.97%; cohort B: 83.01%). But there was a significant reduction in the intensity of CT (mean CT cycles per patient, Cohort A: 4.7; Cohort B: 3.4; $p < 0.01$) and total dose of RT (patients receiving >30 Gy, Cohort A: 51%; Cohort B: 12% $p < 0.001$) administered during this period.
**CONCLUSION:** The survival rates are comparable to the national and international data available. [5-yr survival of patients diagnosed between 1986-1990: Leicester 93%; England & Wales 91%; Scotland 92%, USA 95%; and Europe (1985-89) 90%] (1). The trend towards a better 5-yr survival over this study period has been achieved with less intensive treatment regimens and this should minimise the incidence of long-term treatment related sequelae in these patients with highly curable tumours.

(1) Coleman.1999. Cancer survival trends in England and Wales, 1971 - 1995. Office for National Statistics.

**P104**

**CONFORMAL RADIOTHERAPY FOR PROSTATE CANCER-A SINGLE INSTITUTION EXPERIENCE**

Scrase,C.D.*,Crosbie,J.,Laker,R.,Mackenzie,C.M.,Poynter,A.J.,James,H.V. & LeVay,J.
Department of Clinical Oncology, The Ipswich Hospital NHS Trust, Suffolk, U.K.

**INTRODUCTION**

Randomised data supports the role for conformal radiotherapy in the radical treatment of prostate cancer in achieving comparable tumour control with lower side effects when compared with conventional radiotherapy. In anticipation of these positive results, this institution opted for conformal radiotherapy for all patients to be treated by 3D conformal techniques in addition to those patients enrolled into the MRC RT01 randomised control trial. This programme has evolved over three years now, and we present our experience thus far.

**METHODS**

Retrospective review of all patients treated with conformal radiotherapy with respect to tumour characteristics, use of hormones, treatment technique including outlining of tumour and normal tissue volumes, recorded acute and late toxicity and PSA failure. The RT01 trial definition of PSA>2 and ≥50% rise from nadir was used for PSA failure. The tumour was outlined by the same clinician (CDS) in all but two cases. Inclusion of all or part of the seminal vesicles was determined according to risk formulae. Normal tissues were outlined by the same clinician or at least independently verified. The rectum was outlined according to standardised criteria. Toxicity was scored using the RTOG system.

**RESULTS**

Between 1/99-12/01, 75 patients have been treated. Median follow-up 12.2 months. Clinical stages were T1(37%),T2(55%),T3(8%). PSA ranged from 1.2-114 (median 16). 95% had neoadjuvant hormones, 12% receive adjuvant hormones. 25% received 74Gy. Acute urinary toxicity 32%G2 8%G3. Acute rectal toxicity 40%G2 1.3%G3. Late toxicity thus far (where ≥3mo. follow up) bladder 6%G2 1.5%G3, rectal 10.8%G2 4.5%G3. No patients have developed G4 late toxicity. Late toxicity was predictable from DVH of normal tissues (>55% rectum received ≥95% TD). Latterly, high rectal doses would not be accepted and reduced dosing to the seminal vesicles (50-56Gy) a consideration. Six have biochemical failure. One patient has developed symptomatic local relapse.

**SUMMARY AND CONCLUSIONS**

Our preliminary data supports the decision to adopt conformal radiotherapy as definitive non-surgical treatment in the curative treatment of prostate cancer. The toxicity rate is low and PSA control at this early stage (particularly given the proportion who received neo-adjuvant hormones) favourable and compares well with published data from larger centres. With IMRT being implemented at other tumour sites in this institution, we feel well prepared for its incorporation into the prostate cancer treatment programme and the opportunity to join other dose escalation studies.

© 2002 Cancer Research UK

**P105**
**RADIOTHERAPY FOR BLADDER CANCER**
A. El-Modir*, D. Fairlamb[1]
*Royal Shrewsbury Hospital, Shrewsbury and [1]New Cross Hospital, Wolverhampton, UK

**Objective**: To characterize the patient population referred for radiotherapy for bladder cancer and to assess the effectiveness of radiotherapy in this population.
**Design**: We retrospectively reviewed the medical records of 35 patients referred to the Deanesly Centre at New Cross Hospital between January 1997 and December 1998 for radical radiotherapy for carcinoma of the urinary bladder.
**Material and Methods:** The median age of the 35 study patients was 72 years (range, 51 to 88), and the male:female ratio was 4:1. Two (5.7%) patients had T1 tumours, fifteen (42.8%) patients had T2 tumours, twelve (34.3%) patients had T3 tumours and six (17.2%) patients had T4 tumours. Of the 35 patients, 34 had transitional cell carcinoma and 1 patient had squamous cell carcinoma. Thirty (85.7%) patients had a grade 3 histology. In all patients, the whole bladder was treated with no attempt to treat the pelvic lymph nodes. The median total dose of irradiation was 51.5 Gy.
**Results**: Local control of bladder cancer treated with radiotherapy alone was as follows: T1, two of two patients; T2, fourteen of fifteen; T3, seven of twelve; T4, three of six. Thus, the local control rate was 74%. The actuarial 3-year cause specific survival for T1, T2, T3 and T4 tumours were 100%, 60%, 33% and 17%, respectively. Several other factors, like age, grade, performance status and the presence of ureteric obstruction were important for the outcome. 70% of our patients experienced grade 1 acute intestinal and bladder toxicities, 10-14% had grade 2 toxicities but no grade 3 or 4 acute toxicities were seen. So far, 5 patients developed grade 1 late bladder toxicity and 1 patient developed grade 1 late bowel toxicity.
**Conclusion**: This small series substantiates the effectiveness of radiotherapy in patients with carcinoma of the urinary bladder.

**P106**
**A PHASE I TRIAL OF CONFORMAL RADIOTHERAPY WITH CONCURRENT ONCE-WEEKLY GEMCITABINE FOR INVASIVE BLADDER CANCER**
Sangar VK*, Lyons J, Logue J, Wylie J, Clarke NW & Cowan R.
The Christie Hospital NHS Trust, Manchester, United Kingdom.

This study examines the dose-limiting toxicity of weekly Gemcitabine given concurrently with conformal radiotherapy in bladder cancer.
Eight patients with invasive TCC (T2-3) opting for organ preserving therapy were studied. Treatment comprised conformal radiotherapy (52.50Gy in 20 fractions over 4 weeks) concurrent with once weekly i.v low dose Gemcitabine. The starting dose of 100mg/m$^2$ was escalated in 50mg/m$^2$ increments in successive cohorts. The principles of ICH Good Clinical Practice, the Declaration of Helsinki as well as local regulatory requirements were observed.
Mean patient age was 69 years (8 males). All had invasive TCC (4 T2; 4 T3). Mean pre-treatment IPSS was 5.5 (1-13). Dose-limiting toxicity was observed in two patients receiving 150mg/m$^2$; Grade III Bowel (1) and Urinary (1) toxicity (Mean post-treatment IPSS 11.5). Six patients received 100mg/m$^2$ with no bowel, genitourinary or haematological Grade II-IV toxicity; Mean post-treatment IPSS was 5.5. One of six did not complete Gemcitabine treatment due to a vascular event unrelated to treatment. Of the 8 patients studied, so far 6 (4 at 100mg/m$^2$; 2 at 150mg/m$^2$) have been evaluated for outcome at 3 months by cystoscopy and MR Scan. Five of six showed a CR (3/4 at 100mg/m$^2$; 2/2 at 150mg/m$^2$). One patient (100mg/m$^2$) without a CR underwent salvage cystectomy but died of progressive disease. The remaining patients are well (follow-up 2 – 12 months).
Low dose Gemcitabine (100mg/m$^2$) Chemoradiotherapy is well tolerated and may provide an alternative organ preserving strategy in invasive TCC.

**P107**
**ACUTE MORBIDITY OF HIGH DOSE RATE PROSTATE BRACHYTHERAPY AND THE LEARNING CURVE ASSOCIATED WITH THE DEVELOPMENT OF THIS SERVICE IN THE UK**
McCormack M, Beresford MJ, Birtle AJ, Rickards D, Emberton M, Payne HA. Meyerstein Institute of Oncology, University College Hospitals, London W1T 8AA

Aim  To report the acute morbidity associated with the learning curve for developing a new service of high dose rate brachytherapy for carcinoma of the prostate in the United Kingdom.
Introduction  Carcinoma of the prostate is the second commonest cancer in the UK with approximately 23,000 new cases and 10,000 deaths per year. The treatment options for those with localised disease are surgery or external beam radiotherapy. Despite encouraging results with permanent seed implantation and high dose rate (HDR) afterloading brachytherpay in the USA, the UK has been slow to follow this lead. Our HDR prostate brachytherapy programme was established in 1999. We describe changes in key variables and the associated acute morbidity in the first 15 patients treated.
Methods  Patients with intermediate or high-grade prostate cancer who would benefit from dose escalation were selected. Entry criteria were any of: stage T2a-T3a, Gleason grade >6 or PSA >10, N0, M0. Patients were initially treated with the HDR boost. Applicator needles were inserted under general anaesthetic through a perineal template with the prostate visualised by transrectal ultrasound. The treatment volume was CT-planned using the PlatoTM system and the dose given using an iridium HDR afterloading MicroselectronTM machine. The prescribed HDR boost dose was 16.5 Gy in 3 fractions over 2 days with a minimum interfraction interval of 6 hours. External beam radiotherapy (46 Gy in 23 fractions over 4.5 weeks) was given to a CT planned volume of the prostate and periprostatic tissues.
Results  15 patients with an age range between 57 and 74 years (median age 67 years) were treated with the HDR procedure. 53% had T3a disease. 73% were Gleason grade >8 and 60% had a PSA >20. The learning curve associated with the treatment was steep and was best demonstrated by a 33% reduction for overall treatment time on day one of the HDR procedure. There was also a reduction in time taken for the individual steps in the brachytherapy process (needle implant, CT scanning, planning and treatment delivery). This paralleled the reduction in man-hours for all members of the multidisciplinary team as skills developed. The risk of acute morbidity decreased with experience: reduction in the length of in-patient stay fell from 14 to 3 days, duration of catheterisation fell from 11 to 2 days and duration of haematuria reduced from 10 to 0 days. These factors all varied in proportion to the number of bladder punctures seen at cystoscopy (15 to 0 punctures). Acute bowel toxicity was no different from external beam irradiation alone.
Conclusion  HDR brachytherapy is initially time-consuming, but with a steep learning curve – the duration of the procedure and the associated morbidity are shown to rapidly reduce.

**P108**
**A NOVEL TRI-SPECIFIC ANTISENSE OLIGONUCLEOTIDE (AO) TO THE INHIBITORS OF APOPTOSIS PROTEINS (IAPS) ENHANCES APOPTOSIS IN PC3 PROSTATE CANCER CELLS**
KR McEleny*[1], RWG Watson[1], K Williamson[2], RNT Coffey[1], J Fitzpatrick[1]
[1]Dept of Surgery, Mater Misericordiae Hospital, University College Dublin, 47, Eccles St, Dublin 7, Ireland
[2]Dept of Pathology, Queen's University Belfast

Introduction.
The Inhibitors of Apoptosis (IAPs) block caspase-mediated apoptosis. Prostate cancer cells are inherently resistant to apoptosis and previous studies in our laboratory have demonstrated that the IAPs are over-expressed in prostate cancer. We used a tri-specific AO to c-IAP1, c-IAP2 and XIAP to determine if down-regulation of these IAPs would increase apoptosis in PC3 cells.
Materials and Methods
Time and dose response experiments revealed that 6 hours of incubation with 200nM of oligonucleotide and lipofectin resulted in optimum transfection conditions. These conditions were used to determine the effects on PC3 cells of IAP AO, scrambled AO and lipofectin alone. The end points were: percent apoptosis, (propidium iodide DNA staining using flow cytometry), cell

© 2002 Cancer Research UK

number (Crystal Violet uptake assay) and IAP protein expression (SDS-PAGE western blotting).
Statistical analysis was performed using Student's t-test.

Results Data=Mean ± StandardDeviation.

| | Control | Lipofectin | IAP AO | Scrambled AO |
|---|---|---|---|---|
| % Apoptosis | 3.8 ± 1.3 | 4.7 ± 1.8 | 7.3 ± 2.2* | 4.4 ± 2.0 |
| Cell No. (% Control) | 100 ± 16.2 | 77.9±19.8 | 65.5 ± 26† | 73.0 ± 13.3 |

*p<0.05 v all groups, †p<0.05 v Control only, n=3 independent experiments

The IAP AO significantly induced apoptosis (p=0.002) and reduced cell numbers (p=0.01) compared to untreated controls. Western blotting demonstrated a down regulation in the expression of c-IAP1, c-IAP2 and XIAP in IAP AO treated cells when compared to control cells.
Conclusions
The tri-specific AO enhanced apoptosis in PC3 cells by down-regulating IAP expression. Strategies that target IAP expression, thereby enhancing apoptosis, could lead to the development of new treatments for prostate cancer.

**P109**

**THE EFFECT OF EGF RECEPTOR TYROSINE KINASE INHIBITION USING ZD1839 (IRESSA) COMBINED WITH PHOTODYNAMIC THERAPY IN BLADDER CANCER**

Briggs CH[1,2]*, Betts CD[2], O'Flynn KJ[2], Moore JV[1], Clarke NW[2,3]
[1]Paterson Institute for Cancer Research, Christie Hospital, Manchester
[2]Department of Urology, Hope Hospital, Salford, Manchester
[3]Department of Urology, Christie Hospital, Manchester

INTRODUCTION The EGF receptor is important in TCC and is a target for anti-cancer therapy. The EGFR inhibitor ZD1839 (Iressa) has synergy with radiotherapy in TCC in vitro and reports in other cancers have shown that EGFR is involved in recovery following photodynamic therapy (PDT). This study combines Iressa with ALA-mediated PDT to assess their combined effects on TCC *in vitro*.
METHODS Survival of the bladder TCC line MGH-U1 and its radiosensitive clone S40b was determined following treatment with ALA PDT and Iressa/ALA PDT combinations. Cells were exposed to 5mM ALA with or without Iressa (10mM for 24 hours) during exponential growth. Cells were then exposed to light at 630nm and 20mW power. Experiments were carried out in quintuplicate on at least 3 separate occasions. Flow cytometry determined differences in cellular protoporphyrin IX (the active photosensitiser) and cell cycle between the 2 cell lines following ALA PDT and combination ALA/Iressa PDT, to identify factors which might account for variation in cell survival between the 2 cell lines.
RESULTS Clonogenic survival assays showed that both cell lines were sensitive to PDT alone, though MGH-U1 was significantly more-so than S40b (p<0.001). This was explained by a 4-fold greater level of PpIX fluorescence in the MGH-U1 line. Prior exposure to Iressa produced a 4-fold increase in cell death in the S40b cell line compared with the parent MGH-U1 line (p<0.001). However, there were no significant differences in PpIX fluorescence or cell
cycle between the two cell lines following Iressa exposure which might account for the survival difference.
CONCLUSIONS Iressa (ZD1839) increases the sensitivity of TCC to ALA PDT, though a differential effect is seen in radiosensitive and radioresistant cell lines. This suggests that there may be a role for EGFR inhibition in superficial bladder cancer treatment with photodynamic therapy.

**P110**

**A PHASE II STUDY OF A MODIFIED MVAC REGIMEN IN LOCALLY ADVANCED AND METASTATIC UROTHELIAL CANCERS.**

A. G. Macdonald* and J. D. Bissett, ANCHOR Unit, Aberdeen Royal Infirmary, Aberdeen, U.K. AB25 2ZN.

**Background**. The combination of methotrexate, vinblastine, doxorubicin and cisplatin (MVAC) is a highly effective regimen in the management of locally advanced and metastatic urothelial cancer, with response rates of 39-72%, and median survival times of 10-15 months reported[1,2,3]. The activity of this regimen, however, comes at the expense of considerable toxicity, with grade III/IV neutropenia seen in 80-90% of patients and grade III/IV mucositis seen in 10-22%.
**Objective**. To evaluate a modified MVAC (mMVAC) regimen (designed to reduce toxicity and ease delivery of chemotherapy while maintaining dose intensity) in patients with locally advanced and/or metastatic urothelial cancers, in terms of toxicity, delivered dose intensity, response rate, progression-free survival and overall survival, within a single centre setting.
**Methods**. Twenty-three patients with locally advanced or metastatic transitional cell or squamous cell carcinoma of the urinary tract were treated with a total of 87 cycles of mMVAC between 1996 and 2000. Methotrexate (30 mg/m$^2$), vinblastine (4 mg/m$^2$), doxorubicin (40 mg/m$^2$) and cisplatin (70 mg/m$^2$) were given on day 1 of a 21-day cycle, for a maximum of six cycles, evaluating response after every three cycles.
**Results**. The mean dose intensity for each agent was over 90% of that intended, with six patients requiring dose reduction and nine cycles requiring to be deferred for one week. The regimen was well tolerated with no grade III/IV mucositis seen and only 44% of patients experiencing grade III/IV neutropenia. There were two cases of neutropenic sepsis, but no treatment-related deaths. The overall response rate was 50% (CR rate 15%, PR rate 35%, SD rate 30%), median time to progression was 8.9 months and median survival was 11.9 months. Actuarial estimates for 1-year and 18-month survival are 48% and 42% respectively.
**Conclusions**. Modified MVAC is an easily delivered regimen (necessitating a hospital visit only once every three weeks), which produces lower rates of mucositis and neutropenia than reported for classical MVAC, without obviously compromising response rates and survival in this phase II study.

1. Sternberg CN, Yagoda A, Scher HI *et al* (1989). Cancer 64: 2448
2. Loerher PJ, Einhorn LH, Elson PJ *et al* (1992). J Clin Oncol 10: 1066
3. von der Maase H, Hansen SW, Roberts JT *et al* (2000). J Clin Oncol 17: 3068.

**P111**

### ABSTRACT WITHDRAWN

**P112**

**DO TERMINALLY ILL HAEMATOLOGY PATIENTS RECEIVE MORE INVASIVE TREATMENTS AND LESS PALLIATIVE CARE THAN OTHER PATIENTS?**

Fuller C[1]*, Williams R[1], Stuart NSA[1], Whitaker R[2]
[1]Department of Oncology, Haematology and Palliative Medicine, Ysbyty Gwynedd, Bangor, North Wales, and [2]Institute of Medical & Social Care Research, University of Wales, Bangor, North Wales.

**Background:** Dying haematology patients present specific problems that may not be addressed within the guidelines for care of the dying patient.[1] **Aims**: To determine if haematology patients receive a different pattern of care in the last 12 days of their life compared to those with non-haematological cancer. **Setting:** A combined haematology, oncology, and palliative care unit in a DGH hospital with shared in-patient beds and junior staff. On-site consultants in haematology, medical oncology and palliative care. **Patients and methods:** 75 deaths within the same unit (25 consecutive deaths in 3 patient groups; haematology, oncology and palliative care) were analysed retrospectively from February 2001. The last 12 days of life were examined and the following parameters were assessed: invasive interventions (venous blood sampling; intravenous antibiotics and fluids, chemotherapy; radiotherapy; blood and platelet transfusion and invasive diagnostic tests) and palliative interventions (palliative procedures; use of syringe driver and use of integrated care pathway for the dying patient). **Results:** The haematology patients received more invasive interventions in the last 12 days before death with significant differences in the median number of interventions (13 in haematology group; 4 in each of other 2 groups). However, the analysis failed to show any differences in the number of palliative interventions between the groups. Palliative care patients received slightly more palliative interventions than the other groups, but this did not reach statistical significance. **Conclusions:** In many haematological illnesses the patient would die without intensive supportive treatment which can sustain the patient over months or years. When this treatment is withdrawn the patient quickly deteriorates and death becomes inevitable. Identifying the time when such supportive treatment has become futile or over zealous is difficult. This study indicates that in haematology patients, but not in oncology or palliative care patients, invasive interventions continue until a short time before death. Further research is needed to determine if this is appropriate or is in agreement with the wishes of patients or their relatives [2].

[1] Working Party on Clinical Guidelines in Palliative Care. Changing Gear-Guidelines for Managing the Last Days of Life in Adults 1997-National Council for Hospice and Specialist Palliative Care Services
[2] Stedeford A. An indestructible patient. BMJ 2001; 323:57.

**P113**

**TOXICITY OF TEMOZOLOMIDE WITH RESPONSE MODIFIERS IN METASTATIC MALIGNANT MELANOMA**

P.Lorigan[1]*, S.Danson[2], A.Clamp[2], J.Hodgetts[2], L.Lomax[2], L.Ashcroft[2], M.R.Middleton[2], N.Thatcher[2].
[1]Weston Park Hospital, Sheffield, UK; [2]Christie Hospital, Manchester, UK.

Temozolomide (TMZ) is an imidazotetrazine with a mechanism of action and efficacy similar to dacarbazine (DTIC). It is an ideal candidate for combination regimens as it is well-tolerated and orally bioavailable. This phase II, two-centre study used TMZ alone and combined with interferon-$\alpha$ (IFN) or thalidomide (THAL). 181 patients with metastatic malignant melanoma were randomised to receive six cycles of TMZ 200mg/m$^2$ po eight-hourly for 5 doses q4w, or TMZ 200mg/m$^2$ po od for days 1-5 with IFN 5 megaunits sc three times a week each week q4w, or TMZ 150mg/m$^2$ po (increased after 1 cycle to 200mg/m$^2$ if there was no haematological toxicity) for days 1-5 with THAL 100mg po od q4w. Toxicity data (all and grades 3 and 4) is presented in the table.

| Adverse Events | TMZ alone | | | TMZ-IFN | | | TMZTHAL | | |
|---|---|---|---|---|---|---|---|---|---|
| (% of patients) | all | 3 | 4 | all | 3 | 4 | all | 3 | 4 |
| Nausea | 43 | 4 | 0 | 59 | 4 | 0 | 50 | 6 | 0 |
| Vomiting | 37 | 4 | 0 | 41 | 2 | 0 | 25 | 4 | 0 |
| Constipation | 25 | 0 | 0 | 24 | 0 | 0 | 40 | 0 | 2 |
| Diarrhoea | 12 | 0 | 0 | 13 | 0 | 0 | 2 | 0 | 0 |
| Alopecia | 0 | 0 | 0 | 6 | 0 | 0 | 10 | 0 | 0 |
| Rash | 6 | 0 | 0 | 9 | 0 | 0 | 23 | 2 | 0 |
| Pulmonary | 16 | 0 | 0 | 20 | 6 | 4 | 21 | 2 | 4 |
| Fever | 14 | 0 | 0 | 37# | 2 | 0 | 6 | 2 | 0 |
| Lethargy | 49 | 6 | 0 | 56 | 2 | 0 | 60 | 6 | 0 |
| Per. neuropathy | 0 | 0 | 0 | 0 | 0 | 0 | 0 | 0 | 0 |
| Infection | 20 | 12 | 4 | 17 | 9 | 0 | 12 | 4 | 0 |
| Anaemia | 57 | 8 | 0 | 74# | 2 | 6 | 33 | 2 | 0 |
| Leucopenia | 45 | 20 | 6 | 57 | 13 | 13 | 31 | 12 | 0 |
| Thrombocytopenia | 71 | 31 | 8 | 63 | 20 | 11 | 27 | 10# | |
| 0# | | | | | | | | | |
| Creatinaemia | 10 | 0 | 0 | 13 | 0 | 0 | 10 | 0 | 0 |
| Bilirubin | 14 | 0 | 2 | 7 | 0 | 0 | 6 | 0 | 0 |
| Alk Phos | 39 | 4 | 0 | 26 | 0 | 0 | 25 | 0 | 0 |
| Transaminases | 31 | 2 | 0 | 30 | 2 | 0 | 21 | 6 | 0 |

(# = statistically significant)

Treatment-related performance status was comparable between the three arms. Efficacy data is not yet mature but will be presented at the meeting. Conclusion: Of the 3 regimens tested the combination TMZ-THAL is the least toxic and the most promising.

**P114**

**ASSESSMENT OF RENAL FUNCTION IN OSTEOSARCOMA AND PERIPHERAL PRIMITIVE NEURO ECTODERMAL TUMOUR (PPNET) PATIENTS**

C Siller*, M Burniston[1], A Protheroe, S Picton[2]. Department of Medical Oncology, Cancer Research UK Clinical Centre, [1]Department on Medical Physics, [2]Paediatric Oncology Department, St James's University Hospital, Leeds, LS9 7TF.

Occasionally patients with large solid tumours have very high initial glomerular filtration rate (GFR) as measured by isotope clearance techniques, which, with effective treatment falls to normal range with no obvious explanation. This phenomenon was investigated further because of the importance of GFR to calculate drug dose and monitor renal function. This retrospective study investigated the relationship between the serial GFR assessments made by serum creatinine and isotope clearance techniques.

All patients with osteosarcoma and PPNET diagnosed over a 5-year period in the Paediatric oncology department were studied using a case note review. Treatment schedules, clinical course, isotope GFR, serum creatinine, and monitored episodes of renal dysfunction were documented.

There were 36 patients with a median age at diagnosis of 16.8 years (range 1.6 – 23.6). 20 (56%) patients had a high initial GFR (females >130ml/min/1.73m$^2$ males >150ml./min/1.73ml/min/1.73m$^2$) and 18 of these fell to within normal limits with treatment. There were 12 incidents of acute renal impairment as defined by CTC criteria grade 2 or above. Most of these episodes were attributed to dehydration or to the use of aminoglycoside antibiotics and in almost all cases GFR values subsequently returned to normal.

Overall there was a poorer correlation between serum creatinine and isotope clearance in this group than in other reported populations (r=0.66) but there was a large inter-patient variability in agreement between the two techniques. Large variations in serum creatinine and therefore estimated GFR were noted during periods of intravenous fluid therapy. This highlights the need for careful selection of any serum creatinine assessment subsequently used for drug dose calculation. Samples must be taken whilst a patient is clinically stable and not on treatment.

This audit describes the phenomenon of raised GFR but does not explain the cause. The role of isotope GFR and serum creatinine in monitoring renal function needs to be further defined through prospective studies.

© 2002 Cancer Research UK

**P115**

**RESULTS FROM A PILOT QUESTIONNAIRE OF GFR PRACTICE AMONGST ONCOLOGISTS IN THE YORKSHIRE REGION**

C Siller*, M Burniston[1], A Smith, T Perren, P Selby, A Protheroe.
Department of Medical Oncology, Cancer Research UK Clinical Centre, [1]Department of Medical Physics, St James's University Hospital, Leeds, LS9 7TF.

The assessment of glomerular filtration rate (GFR) in the UK is challenging for cancer physicians. A questionnaire was designed to investigate the practice of GFR assessment within the UK of oncology patients by oncologists, consultants and trainees, both clinical and medical. The questionnaire was first piloted amongst oncologists in Yorkshire. 63 oncologists were invited to participate in the survey. Clinical oncologists were sent a letter with the questionnaire and invited either to fill in the questionnaire and send it back, or to submit it electronically. Medical oncologists were invited by email to submit the form electronically. 30 (48%) replies were received, 16 electronically and 15 by paper. 14 (37%) clinical and 16 (64%) medical oncologists replied. All hospitals in Yorkshire where cancer patients are treated were represented by replies received.

97% of oncologists made at least occasional use of serum creatinine estimates of GFR, with unanimous use of the Cockcroft-Gault algorithm. More patients had GFR assessments by measurement of serum creatinine than by isotope clearance techniques. Perceptions of the different techniques and their accuracies are very variable. Most replies suggested that measurement techniques are very accurate with 83% of responses suggesting errors of 10% or less. Creatinine clearance was suggested as a more accurate technique than serum creatinine by 63% of respondents but estimates of errors for both of these techniques ranged from 5% to 60%.

Most respondents believed that the presence of oedema (97%) and ascites (93%) might lead to problems in the assessment of GFR but few made any comments about action they would take in these circumstances. There was less agreement on the effect of pleural effusions, with only 67% stating that this could influence results. There was no consensus as to whether intravenous fluids could alter GFR.

Most oncologists treat at least some patients with GFR based dosing regimes but a variety of techniques are used – serum creatinine, isotope tracer and creatinine clearance. There is no consensus that different techniques may be appropriate for these patients.

Most oncologists do not routinely drain their patients of any ascitic fluid before administration of carboplatin. The COIN guidelines on management of patients with renal damage do not appear to have been universally accepted with fewer than half of the respondents using them.

Overall GFR measurements or estimates are widely used for patients with cancer. Practices vary considerably, there are no set guidelines as to which patients GFR is performed on or indeed the methods used. A revised GFR questionnaire will be distributed to oncologists nationally.

**P116**

**THE USE OF THE INTERNET AS AN INFORMATION RESOURCE FOR PATIENTS WITH CANCER**

R Simcock, Sussex Oncology Centre, Brighton Health Care NHS Trust, UK

**Introduction** Enormous interest surrounds the use of the Internet as a medium for distributing information regarding healthcare. Many centres have dedicated resources towards providing information on the Internet although there remains comparatively little information on who the chief users of this information are.

**Method** A questionnaire was issued to consecutive new patients registering at the Sussex Oncology Centre. Patients filled out the questionnaire immediately before their first appointment with an Oncologist. Patients were asked to select from a list information resources they had consulted and to rate their perception of the usefulness of those resources. Demographic information and information on access to Internet technology was also requested.

**Results** 79 questionnaires were returned. 38 patients were male and the average age of the group was 65 years (25-82). Only 16 patients (20%) reported personal access to the Internet although a further 29 (37%) reported that their friends or family had access. 94% of the group had received information about their disease from some source. 91% received information from a doctor or nurse, 59% from books and leaflets and 25% from other media. 12 patients (15%) had found information from the Internet, 3 of them despite not having personal access. All patients seeking information from the Internet also sought information from books and leaflets and their doctors. Patients finding information from the Internet rated the usefulness of that information more highly than any other source except the information from doctors. Only one patient found information from the Internet "not useful". 50% of those with personal access to the Internet had not used it to seek information related to their illness. The average age of patients using the Internet was 55 but all age groups were represented (25-82).

**Conclusions** In this group of patients a significant percentage used the Internet to seek information related to their disease and found the information useful however information from doctors was rated as the most useful.

**P117**

**A PROSPECTIVE AUDIT OF PERIPHERALLY INSERTED CENTRAL VENOUS CATHETERS (PICCS).**

MP Decatris[1]*, K Jeffries[1], S Santhanam[1], A Thomas[2], S Khanna[1], S Vasanthan[1], A Benghiat[1], RP Symonds[1], K O'Byrne[1] and WP Steward[1].
[1]Department of Oncology, University Hospitals of Leicester NHS Trust, Leicester LE1 5WW, UK and [2]Department of Oncology, Peterborough Hospital, Peterborough, UK.

**Aim** PICC lines are virtually free from traumatic complications during insertion, easier to maintain than Hickman lines and are increasingly used to administer infusional chemotherapy in our unit. A prospective audit of PICC line insertions has been conducted to evaluate the incidence of complications in Oncology patients.

**Methods** All PICC line insertions for the year 2001 were audited by completing a 6 page-long specifically designed form at the time of insertion including details of the procedure. Information on complications was collected and analysed retrospectively from the patients' medical notes.

**Results** Out of 104 attempts there were 93 (88%) successful line insertions in 88 patients (5 patients had 2 lines). Six lines were inserted under X-ray guidance. Median patient age was 61 (range, 18-80). A median of 2 cycles (range, 0-12) of chemotherapy was delivered during a total of 6,182 catheter-days. The median PICC life was 51+ days (7-203). The main types of chemotherapy delivered were, cisplatin/5-fluorouracil (5-FU) (35%), De Gramont (24%) and oxaliplatin/5-FU (17%). Cannulation was easy in 80% of the cases. There were no major complications from insertion. 70% of patients had no catheter-related complications. Deep vein thrombosis (DVT) was seen in 5 cases needing removal of the line and treatment with Tinzaparin. Exit site infection was seen in 6 cases (5 definite, one possible) needing treatment with oral antibiotics. Coagulase negative staphylococcus septicaemia occurred in one neutropenic patient who required intravenous antibiotics and made a full recovery. 2 cases of systemic infection, possibly related to the lines, were seen; in one patient the line was blocked and removed and in the other cultures were negative but the line was eventually removed.

| Incidence | DVT (Def+Pos) | Infection† (Def+Pos) | Phlebitis (Def+Pos) | Line Blocked | Line Migrated | Fracture |
|---|---|---|---|---|---|---|
| Cases (% of total) | 5+2 (5.4+2.1) | 6+3 (6.5+3.3) | 3+3 (3.3+3.3) | 6 (6.6) | 4 (4.3) | 4 (4.3) |
| /1,000 PICC-days | 1.1+0.3 | 1.0+0.5 | 0.5+0.5 | 1.0 | 0.7 | 0.7 |

Def, definite; Pos, possible; † includes exit site and systemic infection.

**Conclusion** The incidence of catheter-related sepsis and other complications is low in comparison to other published data. The incidence of sepsis isconsiderably lower than that routinely seen with Hickman lines. This audit supports the increasing use of PICC lines for patients needing infusional chemotherapy.

© 2002 Cancer Research UK

**P118**

**ORAL ANTIBIOTICS AND EARLY HOSPITAL DISCHARGE FOR PATIENTS WITH LOW-RISK NEUTROPENIC FEVER WITH COMBINATION: A SINGLE-CENTRE EXPERIENCE**

H Innes*, A Ibrahim, A. Moss and E. Marshall, Clatterbridge Centre for Oncology, Wirral, Merseyside CH63 4JY

Neutropenic sepsis is a potentially life-threatening complication of chemotherapy. In-patient therapy with broad-spectrum intravenous antibiotics has therefore become the standard of care for all patients. However, it is possible to identify criteria by which episodes of 'low-risk' neutropenic fever can be identified. Two large trials[1,2] have demonstrated equivalence for combination oral antibiotics compared with intravenous regimens for in-patient treatment of these 'low-risk' episodes. In addition, we have shown[3] that for such patients oral antibiotics in conjunction with early hospital discharge for patients who remain stable after an initial 24hour monitoring period offers a feasible, safe and more convenient alternative when compared with conventional management.

We have now adopted this approach as standard management of 'low-risk' neutropenic fever in our cancer centre and here report our initial experiences with this policy. We have introduced a standardised care pathway for the treatment of neutropenic fever. Patients are treated with oral antibiotics (ciprofloxacin 750mg bd + co-amoxiclav 625mg tds for 5 days) if they fulfil the following criteria, as judged by the admitting doctor. i) haemodynamically stable, ii) not requiring intravenous fluids, iii) able to tolerate oral antibiotics, iv) absence of a clinically significant focus of infection and v) no allergies to the antibiotics. These patients are then reassessed on the morning following admission and are eligible for discharge if they have remained stable. Patients not fulfilling these criteria are treated with a standard in-patient intravenous regimen (ceftazidime).

To date, 97 episodes of neutropenic fever have been treated according to this pathway. 52 episodes (53.6%) were treated with oral antibiotics. The median stay length for these patients was 36 hours (range 12hrs – 11 days) and 41 of these (78.8%) resolved without changes to the antibiotic regimen or readmission. The remaining 11 episodes were all successfully salvaged without significant sequelae. Of these, only 1 had significant clinical deterioration (within the initial monitoring period) and 3 episodes required changes to antibiotics because of toxicity (vomiting and diarrhoea). Other toxicity was mild. 2 patients (3.8%) required readmission.

In conclusion, our initial experience with this policy support our earlier findings that treatment of low-risk neutropenic fever with oral antibiotics and early hospital discharge is a realistic alternative to standard management. We will present updated results of our experience at the meeting.

[1]Freifield A, Marchigiani D, Walsh T et al. New Eng J Med 341: 305-311, 1999

[2]Kern WV, Cometta A, De Brock R et al. New Eng J Med 341: 312-318, 1999

[3]Davies HE, Smith DB, O'Reilly SM et al. Proc Am Soc Clin Oncol abst 2379, 2000

**P119**

**AGEING & CANCER SUSCEPTIBILITY: IS SYSTEMIC FAILURE OF APOPTOSIS A FACTOR?**

Ruth Gilchrist*, Doug Easton, Emma McKenzie-Edwards, Diana M Barnes, Diana M Eccles, Audrey Ardern-Jones, Shirley V Hodgson, Paula M Duddy, Rosalind A Eeles and Richard S Camplejohn. Richard Dimbleby Dept. Cancer Research, Guys, Kings and St Thomas' School of Medicine, St Thomas Hospital London, SE17EH.

Previously, we reported results of a three year study determining the value of rapid functional assays of p53 status in LFS and LFL families[1]. The Apoptotic Assay measures the apoptotic response of human PBL to ionising radiation and highlights those individuals with a possible p53 defect; patients with p53 mutations are unable to produce an apoptotic response greater than 30%. The final statistical analysis of the study, including breast cancer patients (n=243), controls (n=48) and cancer-free individuals with family history of cancer (n=64), has demonstrated an interesting phenomenon. We report that non-LFS/LFL individuals with breast cancer have a reduced apoptotic response versus controls (adjusted mean difference 5.74, p=0.005). 134 of the 243 female breast cancer patients had radiotherapy or systemic therapy at various times prior to the assay being performed. However, the response for the group of patients receiving no therapy (mean response 40+/-11% SD) was similar to that for the whole group (mean response 39+/-11% SD) and thus, therapy had no significant effect on apoptotic response. Several studies over the past five years report a reduced apoptotic response to DNA damage with increasing age in rodents[2]. Until now, no such data were available in humans. Systemic failure of apoptosis could be one mechanism whereby breast cancer incidence rises sharply to a high constant rate at a genetically determined age[3]. We report a markedly reduced apoptotic response with increasing age in all three sample groups (p=trend for age).

| Age of | Breast | Cancer-free with | Control. (n) |
|---|---|---|---|
| | P=0.001 | p=0.0001 | p=0.04 |
| <30 | 48.9 (6) | 47.9 (7) | 52.4 (19) |
| 30-39 | 42.2 (61) | 44.7 (18) | 48.2 (20) |
| 40-49 | 37.8 (69) | 40.6 (18) | 43.7 (3) |
| 50-59 | 37.6 (76) | 36.1 (14) | 44.3 (5) |
| >60 | 37.1 (31) | 27.9 (7) | 43.7 (1) |

The finding of a significant association between DNA-damage-induced apoptosis and breast cancer risk raises the possibility that apoptotic response is a marker of susceptibility to cancer and could form the basis of a predictive assay.

References.

1. RS Camplejohn, N Sodha, R Gilchrist, ME Lomax, PM Duddy, C Miner, P Alarcon-Gonzalez, DM Barnes and RA Eeles. 2000. British Journal of Cancer. 82(6), 1145.

2. Polyak K, Wu TT. Hamilton SR, Kinzler KW, Vogelstein B. 1997. Cell Death Differentiation. 4, 242.

4. Peto J, Mack T. 2000. Nature Genetics. 26, 411

**P120**

**AN AUDIT OF HYPERBILIRUBINAEMIA (hiB) ASSOCIATED WITH MALIGNANCY.**

F. Nussey * and M. Mackean. Edinburgh Cancer Centre, Western General Hospital, Crewe Road, Edinburgh, EH4 2XU, Scotland.

**Introduction**. A number of patients (pts) with advanced solid tumour malignancy develop symptomatic jaundice within the last few weeks of life. This can be due to intrahepatic disease or obstruction of the biliary tract, for example by porta hepatis lymphadenopathy. Relief of obstructive jaundice in primary biliary malignancy is well established. However, our perception was that a number of pts with obstruction of the biliary tree due to advanced solid tumour malignancy undergo procedures (such as ERCP or PTC) aimed at relieving this with little benefit in terms of symptoms and at the expense of spending many of their last valuable days of life in hospital.

**Aims.** The primary aim of this study was to evaluate the success of interventions performed to relieve the symptoms of hiB in patients attending our centre. Secondary aims included assessment of the number and type of investigations and interventions performed for the investigation and treatment of hiB, as well as examining the length of hospital stay and survival from the diagnosis of hiB.

**Materials and Methods.** Pts were identified from the clinical biochemistry database over the period 1/1/98 to 31/12/98. All pts with a bilirubin recording of greater than 50μmol/l were identified and case notes were examined for those pts known to the oncology directorate. Information relating to age, primary diagnosis, diagnosis of metastatic disease, biochemistry, investigations and interventions was recorded. Survival from the date of hiB was calculated, and the number of nights spent in hospital was obtained from the patient management system. Pts with primary biliary malignancy were excluded. Statistical analysis was performed using Excel 97.

**Results.** 111 pts were identified, and notes were not available for 14. Notes for 98 pts were examined. The mean age was 59.9 years (35-79) and the mean time from primary diagnosis was 29.2 months (1-156). The majority of pts had either colorectal (34%) or breast (28%) primaries. Imaging of the liver was performed in 43% of pts with hiB. 7 of 8 pts had successful procedures performed to relieve hiB, but in 5 of these PTC was required after failed ERCP. The median survival of the intervention group from hiB was median 62.5 days (range 15-180) compared to median 45 days (range 1-395) in the others (p=0.42). Those pts undergoing procedures did however spend

© 2002 Cancer Research UK

significantly more time in hospital (median 27 nights(7-48)) compared to those who did not (median 9 (0-67) ($p<0.05$)).

**Conclusions.** Pts undergoing an intervention to relieve obstruction as a cause of their hiB did not live significantly longer than those pts who did not. They did however spend significantly more of their last weeks of life in hospital.

**Future.** A care pathway designed to standardise investigation and management of these patients will be presented, with the aim of reducing hospital stay. This will be implemented over a one year period and the results will be compared to those from the current audit.

**P121**

**WAITING TIMES AUDIT: ORGANISATION OF ONCOLOGY CLINICS – HAVE WE GOT IT RIGHT?**

J.E Abraham *; L Roberts; R.J Benson; H.M Earl; J Singer. Department of Oncology, Addenbrooke's Hospital NHS Trust, Hills Road, Cambridge CB2 2QQ.

**Introduction;** Patient-centred care should be the basis for organisation of oncology clinics. Have we fundamentally misunderstood what patients perceive to be most important when they attend clinics? The aims of the audit were:

i) To ascertain whether clinic scheduling addresses patients' own priorities.

ii) To assess the influence of disease site or hospital location on clinic requirements.

iii) To compare patients' and doctors' perception of actual consultation length.

**Methods**; A questionnaire format was designed to address the following issues in 5 hospitals within the West Anglia Cancer Network (1 cancer centre and 4 cancer units): **Q1)** How long should your oncologist spend with you for your initial consultation?

**Q2)** How long should your oncologist spend with you for your follow-up consultation? **Q3)** What is an acceptable delay between your diagnosis and initial consultation?

**Q4)** Ideal organisation of oncology clinics would be; **a)** All clinics run punctually, you are seen within 1-2 weeks of diagnosis, but the time is strictly limited to 10-20 minutes. **b)** All clinics run punctually, you have longer consultation times e.g, 30-40 minutes, but there is a longer wait for the initial appointment i.e. 4 weeks. **c)** All clinic consultations are longer e.g. 30-40 minutes, waiting times while in clinic maybe 1-2 hours, but you will be seen within 1-2 weeks of diagnosis. Questionnaires were distributed at registration in reception. The responses were assessed by hospital location and disease site. The study was completed over a 2 week period at each hospital, between 07-10/01.

**Results:** There were 345 assessable patient responses.

**Q1)** and **Q2)** Most patients expected the Oncologist to spend 20 minutes (47%) on the initial consultation and 20 minutes (51%) on follow-up consultations.

**Q3)** The majority expect to be seen within 2weeks of cancer diagnosis (mean value 60%)

**Q4)** The majority (mean value 66%) preferred all clinics to be run punctually, be seen within 1-2 weeks of diagnosis but have shorter consultation times (option **a**). Overbooked clinics were the least popular (option **c**). There was no significant difference in responses seen between the patients with different tumour types or between the different hospitals. Oncologists' actual assessment of consultation duration (which included review of medical notes, radiology and dictation etc) was 5-10 minutes longer, on average, than the patients' assessment.

**Conclusion:** Patients valued shorter waiting times in clinics more than prolonged consultations. Patients' perception of an acceptable initial consultation period (20 minutes) may underestimate the time required to convey all the relevant information. Alternatively a change to the clinic structure or a change in the methods used to convey relevant information may need to be considered. A re-audit including assessment of patient satisfaction regarding consultation length (pre & post consultation) is underway.

**P122**

**EARLY EXPERIENCES OF A REGIONAL RESEARCH NETWORK**

R.I. Harris* & P.Corrie, WACRN, Oncology Centre, Addenbrooke's Hospital, Hills Road, Cambridge CB2 2QQ.

The West Anglia Cancer Research Network (WACRN) was one of the first wave of cancer research networks to be established in 2001 with funding from the National Cancer Research Network (NCRN). The WACRN comprises a cancer centre at Addenbrooke's Hospital (closely linked with Papworth Hospital) and cancer units at Bedford, Harlow, Hinchingbrooke, King's Lynn, Peterborough and Bury St Edmunds. The WACRN serves a population of 1.65 million with approximately 8000 registered cases of cancer *per annum*.

The NCRN funding for trials infrastructure has been used to appoint a Clinical Lead for Research, a Research Network Co-ordinator and a research nurse for each cancer unit. Funds have been allocated for support services such as pharmacy, radiology, pathology and secretarial assistance. Electronic databases to collect and manage trials recruitment data and trial chemotherapy data already existed at the cancer centre and these are being rolled out to the cancer units with master databases maintained at the centre.

In 2000 (the baseline year for the WACRN), 612 (7.6%) patients were recruited into trials; 395 (4.9%) in national, 154 (1.9%) in commercial and 63 (0.8%) in 'in-house' trials. NCRN adopted trials accounted for 279 (3.5%) of these patients. For 2001, the corresponding figures were 542 (6.8%) for all trials, 394 (4.9%) in national, 79 (1.0%) in commercial and 69 (0.9%) in 'in-house' trials with 288 (3.6%) patients in NCRN trials. In each year 29 NCRN adopted trials were actively recruited to in the WACRN.

The presence of research nurses within the cancer units has had an immediate benefit in the quality, ease and timeliness of data collection for the databases and for data management staff at the centre who undertake much of the long term follow-up reporting. At the West Suffolk Hospital, the first to employ an NCRN funded research nurse in April 2001, recruitment is being maintained in the face of closure of high recruiting trials. For the period April - December the number of trials available for accrual doubled. Historically, many patients recruited at the units were referred to the centre for their treatment, however, it is now increasingly possible to offer patients trial entry and associated treatment locally.

The target patient accrual *per annum* into NCRN adopted trials is 800 by April 2004. Accruing 800 patients into NCRN adopted trials alone, whilst maintaining the WACRN's current balance of national, international, commercial and local studies, represents a considerable challenge. Although no increase in accrual to NCRN trials has been realised since April 2001 it is expected that the West Suffolk Hospital example will be repeated in other WACRN units and that after a period of consolidation increases will be seen.

**P123**

**GASTRO-OESOPHAGEAL CANCER AND THE TWO WEEK WAIT: DO THE PROTOCOLS WORK?**

Mr Paras Jethwa*, Dr Ann Keogh, Mr Ian Young[2], Ms Lina Stezhka[2], Mr Michael Hallissey, Mr John Fielding

Dept. of Surgery, University Hospital Birmingham, Queen Elizabeth.

[2]Wolfson Computer Laboratory, University of Birmingham

**AIMS**: To establish the validity of national referral criteria (DOH guidelines) for gastro-oesophageal cancer and adherence to these criteria by primary healthcare physicians.

**METHODS**: A prospective audit of the outcome of referrals made to our centre was performed. A standard referral form was provided to local General Practitioners' that records criteria for direct assess endoscopic referral and allows classification as Urgent or Non Urgent. Our unit uses a computer algorithm to reclassify priority using the criteria supplied on the referral form. All referrals categorised as urgent by their general practitioners were given an appointment within two weeks. General Practitioners' classification was compared with that of the computer algorithm.

**RESULTS**: During the ten month period of our audit 1169 upper GI endoscopies were performed following open access referral.

© 2002 Cancer Research UK

| GP/DOH Guidelines | Total In Group | No. Of New Cancer Cases | % Incidence |
|---|---|---|---|
| Non Urgent/Non Urgent | 253 | 1 | 0.4 |
| Non Urgent/ Urgent | 625 | 17 | 2.7 |
| Urgent/ Non Urgent | 62 | 3 | 4.8 |
| Urgent/ Urgent | 229 | 21 | 9.2 |
| **Total** | **1169** | **42** | **3.6** |

Of the referrals received by our unit primary healthcare physicians incorrectly coded 58.7% of cases. Prioritisation using the guidelines algorithm identified 38 of 42 cancers during this period. Data entry errors resulted in two of the three malignancies identified in the urgent/non urgent group being incorrectly classified. Correct input would have increase pick up in the urgent/urgent group from 9.2% to 10%.
**Conclusions:** Despite initial scepticism adherence to the National criteria for urgent endoscopic referral is associated with a high incidence of diagnosis of malignancy. This study highlights the benefit to the patient of correct classification.

**P124**
**WORKFORCE PLANNING IN BREAST CANCER – A LOOK TO THE FUTURE**

AM Thompson* (1, 3), M Conroy (2), T Crilly (2), G Needham (3).
(1) Department of Surgery and Molecular Oncology, University of Dundee, Dundee DD1 9SY, (2) Conrane Consulting, 116 Pepys Road, London SW20 8NY, (3) Scottish Workforce Planning Group, Scottish Executive, Edinburgh.

The management of women with breast problems, including cancer, is multidisciplinary, involving diagnostic, therapeutic, inpatient and outpatient facilities. The Tayside and Fife regional service has integrated the screening (for women age 50-64) and symptomatic (women of all ages) services and delivers breast services for 360 women with newly diagnosed cancer each year at 2 sites.
**Aim:** to model future workforce requirements based on current practices and projected demand and to examine the effect of changes in working practice.
**Methods:** for workforce planning, a baseline statement of staffing and workload was drawn up in consultation with all staff groups. The current relationship between staffing and workload (productivity) was then derived and the scope for changes in working practice, including improved productivity, within the current establishment laid out. A forecast growth of 20% in breast cancer patients in the service to 2014, (Chetty, 2001), was used to examine the effects on the workforce both for the current productivity ratio and secondly taking into account the changes in roles particularly for breast clinicians and nurses. Projecting the data for Tayside onto a Scottish workforce allows a prediction of the likely increase in workforce required (excluding replacement of current staff retiring or the impact from changes elsewhere in the service).
**Results:** The two major groups requiring expansion are nurses (breast care nurses and non-specialist nurses), where 26 whole time equivalents will be required across Scotland, and doctors where 17 whole time equivalent clinicians in a range of specialties will be needed. How these staff would be distributed across a geographically wide area remains to be seen.
**Discussion:** There are implications for the effects of developing the breast service on other parts of the health service which may be dependent on the same workforce pool in terms of recruitment, training and retention, developing the radiographer and nurse practitioner roles, flexibility for clinical contact roles (skill mix) and management.
These productivity and workload developments take the current practices into account from the point of view of patient and staff numbers, but do not necessarily address the issue of the quality of service delivered. Gradual changes in imaging practices, operating practices, IT developments with their implementation and the delivery of tailored chemotherapy may well significantly influence working practices in the next decade. Quantum changes such as increasing the age range of conventional breast screening for women above 70 years or below 50 years of age are also likely to have a significant impact on workforce requirements.
**Conclusions:** this type of detailed analysis of the current workforce, modelling the effects of changes in working practice and projected increased demands may be useful in service planning in a range of speciailities.

**Reference:** Chetty, U. Breast Cancer. p105-114. In: Scottish Executive Health Department (2001). Cancer Scenarios: an aid to planning cancer services in Scotland in the next decade. Edinburgh, The Scottish Executive.

**P125**
**TRANS-TASMAN RADIATION ONCOLOGY GROUP (TROG) DEVELOPMENT 1989-2002, AND RESULTS OF TRIALS**

Prof Jim Denham* (TROG Group Co-ordinator) Newcastle, NSW, Australia
Dr David Lamb* (TROG Scientific Committee Chair) Wellington, New Zealand
A/Prof David Ball (TROG President) Melbourne, Victoria, Australia
Ms Olga Kovacev (TROG Senior Operations Manager) Newcastle, NSW, Australia

The Trans-Tasman Radiation Group is a research organisation that was founded in 1989 by the presenters and others 'to foster and promote the design and execution of high quality clinical studies which address radiotherapeutic management issues, particularly those that require multi-institutional participation'.
Thirteen years later, a critical assessment is made as to whether or not the group has been successful in achieving this goal.
TROG now has a membership of 92 out of 165 practising radiation oncologists in Australia and New Zealand, and 32 out of 36 radiation treatment centres have at least one TROG member. 3800 patients have been entered into clinical trials.
The important landmarks in the development of the group will be summarised. The main findings of phase III trials will be presented, including some unpublished data. Finally, the future direction of the group will be outlined.

**P126**
**RECRUITMENT OF PATIENTS WITH CANCER INTO RANDOMISED CLINICAL TRIALS: EXPERIENCE AT A SMALL CANCER CENTRE**

A. El-Modir*, R. Agrawal, V. King, M. Adams, H. Moore
Department of Clinical Oncology, Royal Shrewsbury Hospital, Shrewsbury, UK

Background: Although many oncology patients are eligible to participate in clinical trials of cancer treatment, only between 5 and 7% of eligible patients currently enter clinical trials in the UK. Time pressures, lack of funding and support staff, especially in small cancer centres and units, are among the reasons for this.
Methods: From our database, we reviewed all the patients referred to our centre and identified those eligible for entry into clinical trials and whether they have been offered entry into trials. We have also studied the impact of the establishment of a clinical trials unit on our recruitment figures.
Results: 4183 new patients referred to our unit between 1998 and 2001. 723 (17.3%) were deemed eligible for entry into clinical trials. 583 (80.6%) of the eligible patients agreed to enter clinical trials. 94 patients were entered into more than one trial. The majority of patients who declined entry into trials did not state a reason; some patients did not want to have chemotherapy; a small proportion were worried about "randomisation" and few patients stated that they did not want to be "guinea pigs". Our trial recruitment increased over 400% since the establishment of a clinical trials unit with dedicated staff to support clinical trials.
Conclusion: Clinical trials are essential for the evaluation of different cancer treatments.
All oncology centres should establish a clinical trials unit with dedicated staff if recruitment into trials to be increased. The extra costs of clinical trial participation must be recognised.

© 2002 Cancer Research UK

**P127**

**IMPLEMENTING THE SCOTTISH CANCER STRATEGY**

Dr Anna Gregor, Lead Clinician for Cancer Services in Scotland, Mrs Elizabeth Porterfield, Cancer Services Co-ordinator and Elaine Wilson, Implementation Manager* Cancer Team, Scottish Executive, St Andrew's House, Regent Road, Edinburgh EH1 3DG

The Publication of *Cancer in Scotland: Action for Change* signalled a renewed drive to tackle cancer and the causes of cancer in Scotland. An investment of £40 million over 3 years commencing 2001was dedicated to support the implementation of the strategy on top of existing national and local investment plans. The focus of the strategy is on improving diagnosis, treatment and care through innovation and spreading good practice. Three Regional Cancer Advisory Groups (RCAGs) have been created (North, South East and West) to improve the planning and design of local services.

Key features of the strategy include: by October 2001 urgent referrals for breast cancer to begin treatment within one month of diagnosis; by 2005 maximum wait from urgent referral to treatment for all cancers will be two months; managed clinical networks in place for all cancers by 2002, by April 2002 a major programme of service redesign will be in place.

The first RCAG's annual investment and implementation plans were published last November totalling £10.7million. This first wave of investment included funding for more than 130 extra doctors, nurses and other specialist health professionals and vital cancer equipment such as scanners. This was all aimed at building capacity to secure more rapid diagnosis and reduce waiting times, improve access and quality of care and improved access closer to home or at home. But the Cancer Strategy is not just about money it is also about working in partnership and making the best use of the resources that we have.

An implementation framework is being developed to drive forward and monitor the implementation of *Cancer in Scotland*. The RCAGs will report their progress on implementing their investment plans and achieving the targets set out in the strategy on a 6 monthly basis. This presentation reports on the implementation of the first 6 months to March 2002.

**P128**

**AUDIT OF CANCER PATIENT ENTRY TO CLINICAL TRIALS**

J Shaw*, H Cornwell, L Koncewicz, I Inman, P Corrie. Cambridge Cancer Trials Team, Oncology Centre, Addenbrooke's NHS Trust, Cambridge, CB2 2QQ, UK.

A retrospective audit of cancer clinical trial accrual rates was undertaken at Addenbrooke's Oncology Centre. The data presented encompasses new patient referrals for breast, gastrointestinal, ovarian, lung and renal cancers and melanoma to oncologists working in the centre between January 1st and December 31st, 2001. For all these disease sites, regular multi-disciplinary team meetings take place, allowing patients to be screened for eligibility to current clinical trials. Clinicians were responsible for offering patients clinical trial entry at consultation. Our data was obtained from various sources, primarily the disease site specific databases, which hold a limited amount of information on trial accrual. The results are summarised in the table below:

| | Breast | GI | Ovary | Lung | Melanoma | Renal | TOTAL |
|---|---|---|---|---|---|---|---|
| New patient referrals | 408 | 200 | 186 | 81 | 66 | 7 | 948 |
| Patients offered trial entry | 90 | 56 | 12 | 19 | 20 | 2 | 199 (21%) |
| Patients entered | 72 | 35 | 5 | 9 | 16 | 2 | 139 (15%) |
| Patients declined | 18 | 21 | 7 | 10 | 4 | 0 | 60 (6%) |

Our findings that 15% of patients entered a clinical trial compare favourably with similar studies by Spiro et al.[1] and Maughan et al.[2] who reported 11.7% and 4.8% patient accrual, respectively. However, the accuracy of our audit is limited by a failure to capture all pertinent information. For example, we do not know the percentage of potentially eligible patients who were not offered a trial by clinicians, the percentage of patients for whom no trial was available, or the percentage of consenting patients who ultimately failed to meet the trial inclusion criteria (screening failures). Furthermore, we would like to know more about patients who decline trial entry, especially their reasons for doing so. From January 2002, patient databases have been modified and paper proformas implemented to collect additional information to address the shortcomings of this audit. A future audit should help to identify possible barriers, such as trial availability, eligible patients not offered trial entry, and patient perceptions, that need to be addressed in order to improve patient accrual to clinical trials.

1. Spiro SG, Gower NH, Evans MT, Facchini FM, Rudd RM. Thorax 2000; 55:463-5
2. Maughan TS, Branston L, Batt LA, Shankland SH. Br J Cancer 2000; 83 S1:2

**P129**

**THE DETECTION OF EARLY p53 MUTATIONS IN COLORECTAL POLYPS USING THE RESTRICTION SITE MUTATION ASSAY**

*GL Williams[1,2], GJS Jenkins[2], JM Parry[2] and J Beynon[1]
[1]Department of Colorectal Surgery, Singleton Hospital, Swansea, UK
[2]School of Biological Sciences, Swansea University, Swansea, UK.

INTRODUCTION: Most sporadic colorectal carcinomas are derived from precursor lesions known as polyps. This theory correlates well with the molecular biology of neoplastic progression which depends on certain gene mutations. Heavily implicated are APC, K-ras and p53. The tumour suppressor gene p53 is thought to exert its effect late on in polyp progression rather than at initiation. The Restriction Site Mutation (RSM) assay selects mutated sequences at the molecular level hence microdissection of the tissue is unnecessary to enrich the mutant cell population. Mutations of p53 are detectable, even if the cells carrying such mutations are present within an excess of normal cells (sensitivity of 1 mutant sequence amongst 10,000-100,000 normal sequences). The aim of this study was to use the RSM assay to detect whether early p53 mutations were detectable in colorectal polyps.
METHODS: Polyps were obtained from different patients at routine colonoscopy. DNA was extracted by a high salt precipitation method and then amplified by PCR following restriction enzyme digestion by msp1. This enzyme was used to look specifically at one of the major p53mutational hotspots for colorectal neoplasms: codon 248. Following DNA analysis by RSM and RFLP, all suspected mutants were sequenced.
RESULTS: Polyps from 55 patients were analysed for codon 248 mutation. Mutations were found in 4 of the 55 polyps. There were no correlations between these four and severity of dysplasia, histological type, nor with size. Two polyps had a G:A mutation at codon 248, while the other two displayed C:T mutations.
CONCLUSION: We have been able to show with the aid of a very sensitive mutation detection system that mutations of p53 do occur at an early stage in the adenoma to carcinoma sequence. The fact that the RSM assay has detected p53 mutations in different types of polyps may be of benefit to further research into why certain polyps and not others progress to carcinoma. This could become important for patient follow up.

**P130**

**MOST MUTATIONS OF THE APC TUMOUR SUPPRESOR GENE IN SPORADIC COLORECTAL CANCER ARE TRANSVERSIONS**

*GL Williams[1,2], GJS Jenkins[2], JM Parry[2] and J Beynon[1]
[1]Department of Colorectal Surgery, Singleton Hospital, Swansea, UK
[2]School of Biological Sciences, Swansea University, Swansea, UK.

INTRODUCTION: The Adenomatous Polyposis Coli (APC) gene is heavily involved in sporadic colorectal carcinogenesis. APC mutations are the earliest known genetic alterations in progression to colorectal cancer and have been found in aberrant crypt foci and small adenomas. It is therefore no surprise that mutations of APC are thought to initiate the potential path to cancer. Certain types of gene mutations are thought to be due to differing

